# CytoregR inhibits growth and proliferation of human adenocarcinoma cells via induction of apoptosis

**DOI:** 10.1186/1477-3163-5-1

**Published:** 2006-01-09

**Authors:** J Kumi-Diaka, M Hassanhi, J Brown, K Merchant, C Garcia, W Jimenez

**Affiliations:** 1 Department of Biological Sciences, Florida Atlantic University, Davie FL, 33314, USA; 2 School of Medicine, Blood Bank Division, University of Zulia Maracaibo, Venezuela; 3 Cytorex BioSciences Inc. 2700 Glades Circle #138, Weston FL. 3331, USA

## Abstract

**Background:**

Cancer is one of the devastating neovascular diseases that incapacitate so many people the world over. Recent reports from the National Cancer Institute indicate some significant gain therapy and cancer management as seen in the increase in the 5-year survival rate over the past two decades. Although near-perfect cure rate have been reported in the early-stage disease, these data reveal high recurrence rate and serious side effects including second malignancies and fatalities. Most of the currently used anticancer agents are only effective against proliferating cancer cells. Thus attention has been focused on potential anti-cancer agents capable of killing cancer cells independent of the cell cycle state, to ensure effective elimination of most cancer cells. The objective of this study was to test the chemosensitivity and potential mechanism of action of a novel cancer drug, CytoregR, in a panel of human cancer cells.

**Methods:**

the study was performed using a series of bioassays including Trypan blue exclusion, MTS Growth inhibition, LDH-cytotoxicity, TUNEL-Terminal DNA fragmentation Apoptosis Assay, and the Caspase protease CPP32 activity assays.

**Results:**

Cytoreg^R ^induced significant dose- and time-dependent inhibition of growth in all the cells; with significant differences in chemosensitivity (P < 0.05) between the target cells becoming more apparent at 48 hr exposure. CytoregR showed no significant effect on normal cells relative to the tumor cells. Growth inhibition in all the cells was due to induction of apoptosis at lower concentrations of cytoregR (> 1:300). CytoregR-induced caspase protease-3 (CPP32) activation significantly and positively correlated with apoptosis induction and growth inhibition; thus implicating CPP32 as the principal death pathway in cytoregR-induced apoptosis.

**Conclusion:**

CytoregR exerted a dose-and time-dependent growth inhibitory effect in all the target cells through induction of apoptosis via the CPP32 death pathway, independent of hormonal sensitivity of the cells. The present data indicate that not only could CPP32 provide a potential target for regulation of cytoregR-induced apoptosis but also that cytoregR could play a significant role in chemotherapeutic regimen in many human malignant tumors.

## Introduction

Cancer is one of the devastating neovascular diseases that incapacitate so many people the world over. It is one of the top leading causes of death in the western world. Continuous search for cure and prevention over the past years seems to be yielding encouraging results and this gives the impetus for further search for effective therapeutic regimens against this unyielding human and animal diseases. Significant Progress in the fight against human cancer has been made since the early 1960s. Recent reports from the National Cancer Institute [1] and American Cancer Society [2] indicate some significant gain over the scorch of cancer as seen in the increase in the 5-year survival rate over the past two decades. The overall cancer incidence rates dropped 0.5 percent per year from 1991 to 2001, while death rates from all cancers combined dropped 1.1 percent per year from 1993 to 2001 [1]. Death rates from all cancers combined have been decreasing since the early 1990s. Death rates decreased for 11 of the top 15 cancers in men, and eight of the top 15 cancers in women. Lung cancer deaths rates among women leveled off for the first time between 1995 and 2001, after continuously increasing for many decades [1]. This year's report indicate that between the periods 1975–1979 and 1995–2000 the five-year cancer survival rate improved for most of the top 15 cancers in both men and women, and the top ten sites in children [1]. However, the incidence rate of Breast cancer in women is among the cancers that are still rising. And in men, although significant gains are made in the survival rates in many cancers including the prostate, the incidence rate in prostate cancer is rising [1]. Furthermore, the incidence of testicular cancer has increased three-folds over the past 40 years especially in the Caucasian young males in the 15 to 35-age range [3-5], in spite of high sensitivity of this cancer to treatment. The risk of developing testicular cancer in a man's lifetime is approximately 1 in 500 occurring in approximately 1 in 25,000 men per year [2]. Testicular cancer is 4 times more common in Caucasians than in Afro-American men, with intermediate incidence rates among Native Americans, Hispanics and Asians [6,7]. It is the third (14.3% of all male cancers) most commonly diagnosed cancer among males aged 15–19 years [8]. Testicular cancer has shown the highest cure rates of all human cancers. Most cancer patients, especially those diagnosed at the early stage, can now expect to survive the disease [9,10]. Satisfactory survival rate is being achieved in advanced metastatic disease by the use of multidisciplinary therapeutic approach [9,11]. Although near-perfect cure rate with chemotherapy have been reported in early-stage disease [10], these results reveal high recurrence rate, serious side effects [11-13], and disease progression (second malignancies) and treatment-induced fatalities.

Most of the currently used anticancer agents are only effective against proliferating cancer cells [22]. This partially accounts for the high post-treatment recurrence rate of testis cancer. Currently, attention is being focused on potential anti-cancer agents capable of inducing apoptosis cell death independent of the cell cycle state, to ensure effective elimination of all/most cancer cells. One such reagent of interest is the novel drug, cytoregR

Cytoreg^® ^is a novel anticancer drug candidate which uses hydrofluoric and sulfuric acids as active compounds. The product is a pharmacologically active low pH acid mixture (pH < 1.0) of which hydrofluoric acid (HF) and sulfuric acid (H_2_SO_4_) are active principles. The fluoride ion in cytoreg induces the synthesis of interleukins-1 and 6 (IL-1, IL-6), tumor necrotic factor alpha (TNFα), and stimulating factors of macrophages and granulocytes. IL-1 activates T cells and other cytokines. IL-1 is known to be chemo-protective and radiosensitive, protecting animals against myelosuppression. Cytorex is focused on developingcompounds to treat cancer and immunological-based diseases. Cytorex has world wide intellectual property rights to Cytoreg^® ^and CytoregUNO™ [30].

Cytoreg^® ^has demonstrated anticancer activity in a battery human cancer cells in *in vitro *studies in our laboratory, and limited preliminary pre-clinical studies elsewhere. Several preliminary in vitro studies in our laboratory indicate that cytoregR inhibits growth and proliferation through induction of apoptosis in a battery of human cancer cells. In this study we hypothesized that the cytoregR will induce growth inhibition of human prostate, breast and testis cancer cells through induction of apoptosis. The goal of this study was to assess degree of potential chemosensitivity of cytoregR to these cancer cells and mechanism of action of the drug in the target cells.

## Materials and methods

### Chemicals

Culture media (RPMI 1640 complete), antibiotics, fetal bovine serum (FBS), trypsin-EDTA, and other culture materials were purchased from Sigma Scientific (St. Louis, MO, USA). The caspase kit (Ac-DEV-AFC/z-VAD-fmk) was from Geno Tech Inc. St. Louis MO.

### Test agents

Cytoreg^® ^was supplied by Cytorex Biosciences, Inc.

### Cell lines

GI-101, an estrogen-independent metastatic human breast cancer cells, was obtained from Rambaugh-Goodwin Cancer Research institute (RGI) at Plantation FL; MCF-7, estrogen-dependent breast cancer cells, were initially obtained from the American Type Culture Collection (ATCC) and maintained in our laboratory; PC3, androgen-dependent human prostate adenocarcinoma cells, were originally obtained from the ATCC and maintained in our laboratory. Other human cancer cells originally obtained from ATCC were: LNCaP, androgen-independent adenocarcinoma of the prostate; INTERA-2 cl.D1, androgen-dependent human adenocarcinoma cells of the testis; and Hs 1.Tes, normal human testis cell lines (nPL) Normal primary human placenta cells were a courtesy of Prof Manzur Hassanhi (Medical School at University of Zulia, Maracaibo Venezuela). All cells were cultured and maintained in humidified atmosphere at 37°C and 5% CO_2 _in RPMI1640 medium supplemented with 10% bovine fetal serum (FBS), 2 mM glutamine and 100 U/mL of penicillin + 100 mg/mL of streptomycin (Sigma, St. Louis, MO. USA).

### Culturing and drug treatment of cells

Cells were plated/dispensed into wells of 96-well microtiter plates (MTP) at a concentration of 2 × 10^3 ^cells/well in 100 μl volumes, and incubated for 48 hrs at 37°C in a humidified atmosphere containing 5% CO_2 _to allow adherence, prior to exposure to varying concentrations/dilutions of cytoregR (2000, 1000, 500, 200, 100, 50 dilutions; the highest dilution being the lowest exposure concentration, and the lowest dilution being the highest exposure concentration). Briefly, the cells were treated with the predetermined concentrations in triplicates and cultured/grown in humidified atmosphere at 37°C and 5% CO_2 _for up to 48 hr. post-treatment. After the 48 hr incubation, the supernatants were collected and frozen for lactate dehydrogenase (LDH) determination; the adherent cells were evaluated for post-treatment viability using Trypan blue exclusion and MST assays. Information on cell numbers relative to controls was important in order to determine whether cytoregR was toxic or simply slowing or inhibiting cell replication. The TUNEL assay was used to determine treatment-induced apoptosis in the cells; and Caspase-3 protease involvement in the cytoregR-induced apoptosis was evaluated as described below: The experiment was repeated twice with the same dose ranges to quantify growth inhibitory activity.

#### MTS growth inhibition assay

MTS is a calorimetric assay based on the ability of viable cells to convert MTS to formazan; the quantity of formazan product, as measured by 490 nm absorbance is directly proportional to the number of viable cells in culture. Briefly, at the end of the 48 hr incubation period with the drugs as described above, 100 μl of the growth medium was removed. The cells were then incubated with 20 μl MTS tetrazolium compound for 3 hr at 37°C. During the incubation the MTS salt is metabolically reduced only by viable cells into an insoluble colored formazan; and the absorbance/optical density (OD) was read and recorded on a Multiscan ELISA MTP reader at a single wavelength of 490 nm. The data were presented as percent post-treatment recovery (%live cells) where the absorbance of from the control, non-treated cells was defined as 100% live cells. The % recovery (%Live cells) were graphed (Y-axis) against the concentrations (X-axis) where IC_50 _values could be interpolated from the graph.

#### LDH-cytotoxicity assay

Lactate dehydrogenase activity was measured by a non-radioactive protocol using the LDH cytotox kit (Cat. #. 1644 793: Boehringer-Mannheim GmbG, Bochemica). *Test principle*: The LDH assay is based on the release of the cytosol enzyme, lactate dehydrogenase (LDH) from cells with damaged cellular membranes. Thus in cell culture, the course of drug-induced cytotoxicity can be followed quantitatively by measuring the activity of LDH in the supernatant. Methodology: The previously frozen supernatants were thawed for LDH determination. Briefly, 100 μL/well of each cell-free supernatant was transferred in triplicates into wells in a 96-well microtiter plates (MTP) and 100 μL of LDH-assay reaction mixture (Kit: dye-catalyst mixture) added to each well. After 3 hr incubation under standard conditions of 37°C 5% CO_2_, the absorbance/optical density of the color generated was read on Multiskan biochromatic automatic microplate reader at 490 nm. Percentage treatment-induced cytotoxicity was computed as follows:



Where, OD_t _= mean optical density of treated cells; OD_c _= mean optical density of controls without treatment.

The ODs were graphed (Y-axis) against the concentrations (X-axis) and the IC_50 _values interpolated from the graph

#### TUNEL-Terminal DNA fragmentation apoptosis assay

The presence of apoptosis was determined by terminal deoxynucleotidyl transferase (TdT)-mediated dUTP nick end labeling (TUNEL), using the ApopTag^R ^kit (Boehringer Mannheim Co, Indianapolis, IN) as previously described (14). The kit reagents detect apoptotic cells *in situ *by specific end labeling and detection of DNA fragments produced by the apoptotic process. Briefly, cells were suspended in phosphate buffered saline (PBS) and aliquots were first pre-stained with supra-vital propidium iodide (PI) stain, and examined under fluorescence microscope to differentiate between apoptosis (PI negative) and necrosis (PI positive), according to previously described procedure [15]. To perform the TUNEL assay, slides were prepared with aliquots from the PBS-cell suspensions. The cells (slides) were then permeabilized with Triton X-100 at 4°C for 2 min; then flooded with TdT enzyme and digoxigenin-dUTP reaction buffer (TUNEL) reagent for 60 min at 37°C; followed by further incubation in anti-fluorescein antibody Fab (conjugated with alkaline phosphatase). After this the cells were stained with chromogenic substrate (Fast Red) and mounted. Negative controls were performed by substituting distilled water for TdT enzyme in the preparation of working solutions. The stained mounted cells were examined at 100×, 200× and 400× magnification of the microscope (Olympus BH-2). Cell death was quantified by counting 150 cells in 5–10 separate fields of view per slide, and noting the percentage of apoptotic cells based on morphological appearance, as previously described [16,17]. Percentage treatment-induced apoptosis was computed as follows:



#### Caspase protease CPP32 activity

The activity of caspase protease-3 (CPP32) was determined by using a fluorescent substrate for caspase-like proteases (Apo-Assay kit containing CPP32 specific peptide, Ac-DEV-AFC, and CPP32 inhibitor, zVAD-fmk, + lysing buffer: Geno Tech Inc. St. Louis MO). The cells were preincubated with and without caspase inhibitor, z-VAD-fmk, prior to treatment with genistein isoflavone. Cells were then assayed for caspase protease activity. Briefly, 1 × 10^3 ^cells/ml were seeded in triplicates in 96-well microtitre plates, cultured for 48 hr as previously described. Caspase inhibitor, z-VAD-fmk (10 μM) was directly dispensed into each well and incubated for 2 hr. The supernatants were carefully aspirated and discarded; and the cells treated with varying concentrations of genistein (G_1–70_) for up to 48 hr; harvested and centrifuged at 10,000 rpm for 10 min, then resuspended in PBS. One half of each cell suspension was processed for apoptosis determination. The other half was centrifuged again and the pelleted cells resuspended in hypotonic lysing buffer on ice. After 30 min on ice cells were disrupted by passing 20 times through 27 G needle and centrifuged at 10,000 rpm × 10 min. The supernatants (25 – 50μg total protein) were incubated with caspase reagents for 2 hr at 37°C, and substrate cleavage determined by recording the absorbance at 405 nm on ELISA plate reader as previously described [18,19] and caspase-3 (CPP32) activity was expressed/calculated as a percentage of the positive control. In principle, the level of caspase activity in the cell lysate is directly proportional to the color reaction; hence to the degree of apoptosis.

#### Statistical analysis

The experiments were performed in triplicates and repeated twice, to confirm similar results. Significance of the differences in mean values was determined using the Student's t-test and considering P < 0.05 to be statistically significant [31]

## Results

### Cell growth inhibition

**Figure 1:**
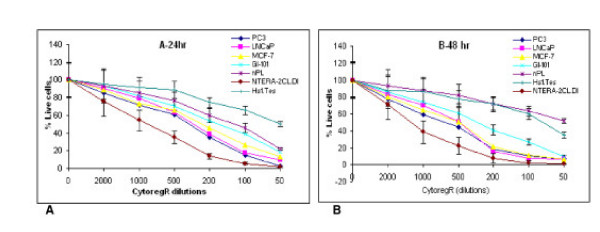
**Growth inhibition of human adenocarcinoma cells**. Cells were grown and treated with indicated concentrations of cytoregR as described in the methods. Cell growth inhibition (cell death) was assessed by passage of Trypan blue dye into the cells, observed under microscope. The percentage of the live cells was determined by as described in the methods. **A-24 hr: **Percentage of live cells after 24 hr treatment. **B-48 hr: **The percentage of live cells at 48 hr treatment. The values represent the means +SEM of triplicates from two independent experiments. Bars = SEM

**Figure 2:**
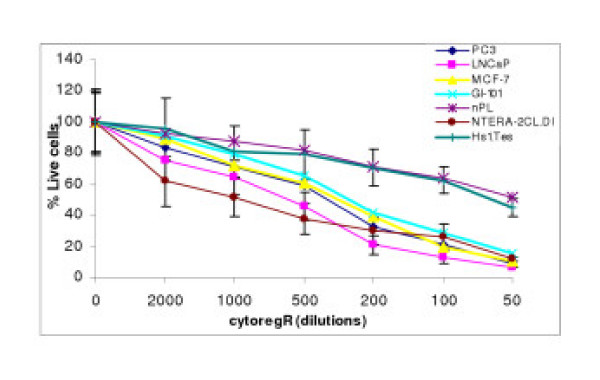
Growth inhibition of human adenocarcinoma cells. Cells were grown and treated with cytoregR for up to 48 hr as described in the methods Cell viability was assessed by the ability of live cells to metabolically reduce MTS Cell viability was assessed by the ability of live cells to metabolicallyreduce MTS to purple formazan after treatment with cytoregR for 48 hr. Percentage viability was determined by setting the absorbance of control, non-treated cells as 100%. Values represent the means + SEM of triplicates from two independent experiments Bars = SEM

**Figure 3:**
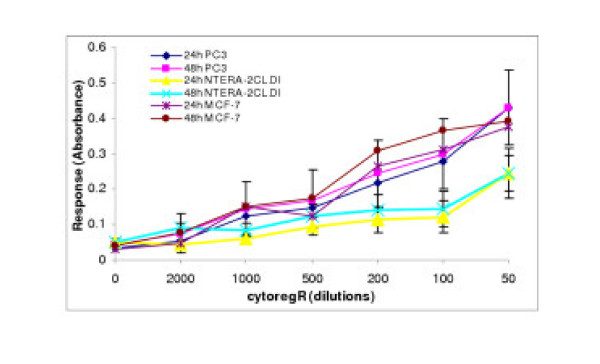
Growth inhibition of human adenocarcinoma cells exposed to cytoregR. Cells were grown and treated with indicate concentrations/dilutions of cytoregR as described in themethods. Treatment-induced cytotoxicity was assessed by measuring the release of lactate dehydrogenase from dead cells, using the LDH assay as described in the methods. The values/data points represent the means ± SEM of triplicates from two independent experiments

**Figure 4:**
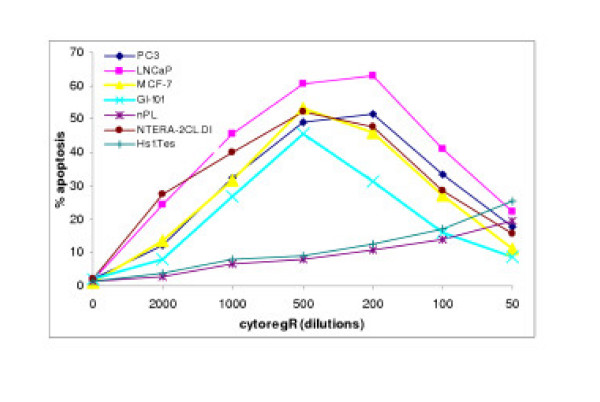
Treatment-induced apoptosis in human adenocarcinoma cells Cells were grown and treated with indicate concentrations/dilutions of cytoregR as described in the methods. Percentage of apoptosis was determined by the TUNEL DNA fragmentation assay as described in the methods. The values represent the means ± SEM of triplicates from two independent experiments.

Cytoreg^® ^was examined for its ability to inhibit growth and proliferation in a panel of human cancer cells [breast cancer cells – GI-101, MCF-7; prostate cancer cells – PC3, LNCaP; testis cancer cells – INTERA-2 cl.D1; normal human placenta – nPL; normal human primary testis cell line – Hs 1.Tes]. Cytoreg^R ^was added to the adherent cells directly at dilutions of 50, 100, 200, 500, 1000, and 2000 as described in the materials. The data obtained revealed that Cytoreg^R ^induced significant dose- and time-dependent inhibition of growth and proliferation in all the cells with significant differences in chemosensitivity (P < 0.05 – P < 0.01) in the different cancer cells as summarized in Figs 1, 2, 3 and Table 1. Difference in chemosensitivity to cytoregR increased with exposure time, more apparent at 48 hr incubation. The Trypan blue exclusion stain indicated that cell viability significantly decreased in all cell types with increasing exposure concentration of the Cytoreg^R ^but no significant effect on the normal cells relative to the tumor cells at the physiological pH7.1 (the 1:500 dilution) of Cytoreg^R ^[Figs 1, 2, 3, 4]. Significant negative correlations were found between LDH and MST & Trypan blue activities (r = -.75, P < 0.01), and significant positive correlations between MTS and Trypan blue exclusion assays (r = 63, P < 0.01). These results indicate the validity/accuracy of the data obtained. Significant treatment-induced morphological changes indicative of cell death and growth inhibition were observed in all the cells with increasing concentration of cytoregR.

**Table 1:** Effect of cytoregR on cancer cells at the dilution of 1:500 (pH7.1) and 48 hr incubation/exposure

**Assays**	**PC3**	**LNCaP**	**MCF-7**	**GI-101**	**nPL**	**NTERA-2CL.DI**	**Hs1.Tes**
**T/blue**	44.06(6.5)^a^	51.08(7.9)^b^	51.08(8.3)^b^	61.01(11.6)^c^	81.89(13.5)^e^	22.08(3.9)^f^	77.56(13.7)^e^
**MTT**	59.25(10.8)^a^	45.85(6.6)^b^	60.88(11.1)^a^	65.12(13.2)^c^	81.89(14.7)^d^	37.57(5.5)^e^	79.25(12.9)^d^
**LDH**	0.167(.03)^a^	0.196(.07)^b^	0.175(.04)^c^	0.193(.07)^b^	0.061(.01)^e^	0.125(0.06)^f^	0.073(0.01)^g^
**%apopt**	49.19(8.8)a	60.76(11.7)^b^	53.15(9.5)^c^	45.59(7.4)^a^	8.05(0.75)^d^	52.25(7.9)^c^	9.15(1.9)^e^
**%CPP32**	35.04(4.9)^a^	41.08(5.8)^b^	37.35(6.6)^a^	28.85(6.1)^c^	9.56(0.93)^d^	33.65(4.5)^a^	11.06(2.3)^d^

% apopt = % apoptosis; %CPP32 = %caspase-3 protease; T/blue = Trypan blue exclusion assay.

Numbers in brackets () are standard errors of means (SEM): Rows with different superscripts are statistically different (P < 0.01)

### Treatment-induced apoptosis

**Figure 5:**
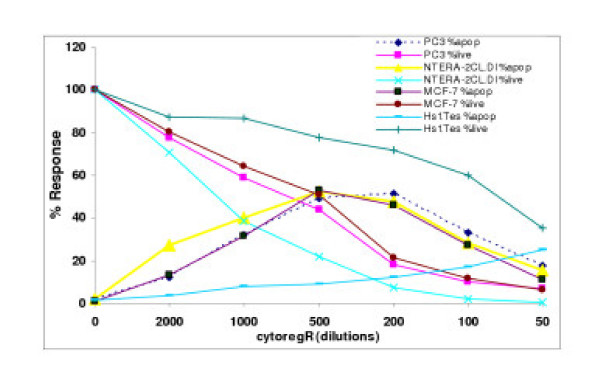
Correlation of % viable cells as assessed by the Trypan blue exclusion assay and apoptosis in treated cells. Percentage apoptotic cells was determined by the TUNEL DNA fragmentation assay as described. The treatment-induced reduction in viable cells corresponded with simultaneous increase in apoptotic cells at corresponding dilutions/concentrations of cytoregR. The data points represent the means ± SEM of triplicates from two independent experiments.

**Figure 7:**
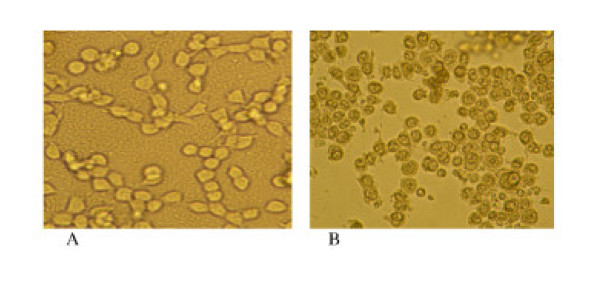
Photomicrographic changes in human adenocarcinoma cells, LNCaP, characteristic of apoptosis; becoming more evident after 48 hr treatment with 1:500 dilution of cytoregR. A: control LNCaP cells without treatment and incubatingfor 48 hr; cells showing normal morphological characteristics of proliferation. B: Light photomicrograph of LNCaP cells after 48 hr treatment; showing cell shrinkage, nuclear condensation and fragmentation -classical characteristics of apoptosis.

The TUNEL DNA fragmentation analysis was used to determine mode of cell death in growth inhibition in the treated cells as described in the methods. Figs 4, 5 and Table 1 revealed that CytoregR induced apoptosis as the main mode of growth inhibition in the panel of cells studied; in a dose-dependent manner, and with significant differences in chemosensitivity (P < 0.01) between the cells. The normal, non-cancer cells, nPL and Hs 1.Tes testis cells, were not significantly affected (P < 0.05) by CytoregR at the physiological pH7.1 (1:500 dilution). However, with increasing exposure concentrations (lower dilutions, 200 – 50) cell death due to necrosis significantly increased concurrent with decreasing apoptotic cell death (Figs 4, 5, Table 1). The classical characteristics of apoptosis – cell shrinkage, nuclear condensation, formation of apoptotic bodies-were observed in all cells (Fig 7-microphotos), to varying degrees; indicating that growth inhibition was predominantly due to treatment-induced apoptosis.

To determine the relationship between growth inhibition and apoptosis induction, we correlated the percentage of viable cells with the percentage of induced apoptosis. Fig 5 revealed that decreasing viability (increasing growth inhibition) concurrently paralleled increasing rate of apoptosis induction in the cells. The overall data revealed a significant negative correlation between MTS/Trypan blue exclusion and apoptosis induction (r = -0.67, P < 0.01).

**Figure 6:**
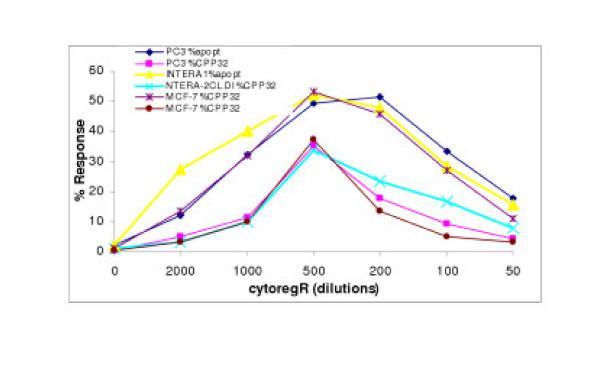
Correlation of cytoregR-induced expression of caspase-3 protease (CPP32) and apoptosis in adenocarcinoma cells. Cells were grown and treated with varying dilutions of cytoregR as described in the methods. Caspase-3 protease activation was determined by using a fluorescent substrate for caspase-like proteases as described in the methods. CPP32 expression correlated positively with percentage of apoptosis induction. Data points represent the means ± SEM of triplicates from two independent experiments.

To determine the potential involvement in the caspase protease 3 in the molecular mechanism of Cytoreg^R^-induced apoptosis, the cells were treated with Cytoreg^R ^co-administered with or without CPP32 inhibitor (DEVD-fmk), and then cultured as previously described. Post-treatment apoptosis was determined as previously described. CytoregR induces expression of CPP32 in all the cells with subsequent induction of apoptosis; consistent with previous observations in genistein-treated cells (Kumi-Diaka et al, NA Sanderson and Alexis Hall, 2000). As shown in Fig 6, treatment-induced expression of caspase-3 protease (CPP32) significantly correlated with treatment-induced apoptosis in all the cells, with manifestation of dose dependency on Cytoreg^R^. It was observed that blocking of CPP32 with the caspase-3 inhibitor, DEVD-fmk, significantly (P < 0.001) reduced apoptosis induction in the cancerous cells; indicating the significant role of CPP32 in the molecular pathway of Cytoreg^R^-induced apoptosis in the panel of cells studied, consistent with observations in other previous studies. Blocking the expression of CPP32 did not completely stop treatment-induced apoptosis in any cell; suggesting the existence of an alternative or potential, caspase-independent death pathway in Cytoreg^R^-induced apoptosis in all the cells. Caspase protease-3 activation/expression significantly correlated with apoptosis induction (0.78, P < 0.01).

## Discussions

The battery of assays that were used in the present studies indicates that cytoregR inhibits cell growth and proliferation in the panel of human cancer cells through induction of apoptosis at the physiological concentration/dilution of the drug. Growth inhibition of the tested cells strongly correlated with the MTT, Trypan blue exclusion, and LDH assay results. The pattern of response in all the targeted cells was significantly similar, and consistent with results of previous studies with genistein isoflavone [18,20,21]. The treatment-induced morphological changes observed in all the cells (indicative of cell death, and growth inhibition) were consistent with previous observations in other studies with other cytotoxic agents [18,22]. The inverse correlation between the MTT and the LDH assays offers a strong support for the reliability of the data. The overall data demonstrate that Cytoreg^R ^significantly inhibit cell growth and proliferation through induction of apoptosis as the main death pathway in all the cells; consistent with previous results in with other agents [23,24]. In all the cells, induction of apoptosis correlated inversely with decrease cell viability, concurrent with increasing concentration of cytoreg^R^; further indicating that apoptosis was mostly accountable for cell death and growth inhibition. The observed bio-morphological changes indicative of apoptosis were identical in pattern and severity in the cells; consistent with previous results [25,26]. However, cytoregR had no significant effect on normal, non-cancerous human cells relative to the cancer cells studied. The bio-morphological hallmarks of apoptosis observed in the treated cells were in conformity with previous observations in apoptosis in other mammalian cancer cells [26,27]. Similar to observations in preliminary studies, the chemotoxic effects of cytoreg^R ^in the cells at dilutions within physiologic (pH 7–7.2) concentrations (dilutions of 2000, 1000, 500, 200) were selective for the cancer cells studied; thus indicating that the differential effects of cytoreg^R ^on the human tumor versus normal cells is mostly due to the chemical composition of the compound and not the acidity of the compound.

The potential signaling pathways in cytoregR-induced apoptosis is under investigation presently. However, in the present results the significant positive correlation of CPP32 expression with percent apoptosis induction in all the cells strongly implicates caspase-3 in the apoptosis inducing mechanism in the cells. The observation is consistent with the significant role of caspase-3 protease in genistein-induced apoptosis in both human testis and prostate cancer cells [18,19,21,28,29].

CytoregR induced apoptosis in both hormone-sensitive (GI-101, LNCaP) and hormone-independent cancer cells (PC3) to varying degrees; with significant differences in sensitivity. Therefore cytoregR could be used in treatment of cancer cells independently of hormonal sensitivity of the cells. As observed in this study, since both hormone-sensitive and hormone independent tumors were susceptive to growth inhibition by cytoregR, it could be inferred that the action of cytoregR does not appear related to hormone modulation/ regulation activities, at least not directly.

## Conclusion

It is concluded from the data obtained that: i) Cytoreg^R ^exerts a dose- and time-dependent growth inhibition effects in the panel of the cancer cells investigated, and with significant differences (P > 0.05)in chemosensitivity between the cells; ii) growth inhibition in cells was mostly through the apoptosis death pathway; iii) CytoregR did not significantly inhibit growth in the normal placenta and testis cells studied; iv) Cytoreg^R ^induces activation/expression of CPP32 concurrent with apoptosis induction in all the cells; thus implicating utilization of CPP32 in the apoptosis-induction death machinery; consistent with previous data; and v) caspase-3 protease could provide potential target for regulation of Cytoreg^R^-induced apoptosis in the panel of cells investigated independent of hormonal sensitivity of the cells. Our data show that Cytoreg^R ^is a strong inhibitor of proliferation and growth of a series of human cancer cells, and thus supports the view that this novel drug could play a significant role in chemotherapeutic regimen for at least some human malignant tumors. Preliminary in vivo animal studies and limited human studies showed no significant side effects.

## Competing interests

We the authors, declare that we have no competing interests of the sort with respect to this research.

## Authors' contributions

JK-D contributed to the conception, and design of the experiment; contributed to the culture, the bioassays, analysis of the data and initial writing/drafting of the manuscript. HM contributed to the culturing and bioassays, to the collection of data and helped in the final writing of the manuscript. CG and WH participated in the design of the study, collection and statistical analysis of the data. JB and KM helped in the culturing and bioassays (independently) and prove-read the final manuscript for submission.
